# Supplementary material for: Hybrid Models and Biological Model Reduction with PyDSTool
Source: PLoS Comput Biol. 2012 Aug 9;8(8):e1002628. doi: 10.1371/journal.pcbi.1002628 (PMC3415397; doi:10.1371/journal.pcbi.1002628)
Supplement: Text S4 — Complete source code for the PyDSTool package (version 0.88.120504). Includes API documentation and help files linking to web pages. This file is identical to the current public release on Sourceforge.net. (ZIP) [file pcbi.1002628.s004.zip › PyDSTool/html/PyDSTool.Toolbox.optimizers-module.html]

xml version="1.0" encoding="ascii"?


PyDSTool.Toolbox.optimizers


| Home | Trees | Indices | Help | PyDSTool |
| --- | --- | --- | --- | --- |

Package PyDSTool ::
Package Toolbox ::
Package optimizers

[hide private]

[frames] | no frames]

# Package optimizers

source code

Optimization module


|  |  |
| --- | --- |
| Submodules | [hide private] |

- **PyDSTool.Toolbox.optimizers.criterion**: *Module containing every criteria for converge test*
  - **PyDSTool.Toolbox.optimizers.criterion.composite\_criteria**: *Composite criteria allow to use several criteria together, with
    and/or composition*
  - **PyDSTool.Toolbox.optimizers.criterion.criteria**: *A list of standard convergence criteria based on the number of
    iterations, the last values taken by the cost function and the
    associated points*
  - **PyDSTool.Toolbox.optimizers.criterion.facilities**: *Proposes a way to create a composite criterion*
  - **PyDSTool.Toolbox.optimizers.criterion.information\_criteria**
- **PyDSTool.Toolbox.optimizers.defaults**: *Defines the defaults parameters for the generic optimizer framework*
- **PyDSTool.Toolbox.optimizers.helpers**: *Helper functions*
  - **PyDSTool.Toolbox.optimizers.helpers.finite\_difference**
  - **PyDSTool.Toolbox.optimizers.helpers.levenberg\_marquardt**
  - **PyDSTool.Toolbox.optimizers.helpers.quadratic**
- **PyDSTool.Toolbox.optimizers.line\_search**: *Module containing the line searchers*
  - **PyDSTool.Toolbox.optimizers.line\_search.adaptive\_last\_step\_modifier**: *Line search decorator that overrides the default alpha\_step value
    with a factor times the last alpha\_step, the factor being a
    function of the current direction and the last direction*
  - **PyDSTool.Toolbox.optimizers.line\_search.backtracking\_search**
  - **PyDSTool.Toolbox.optimizers.line\_search.barzilai\_borwein\_non\_monotone\_search**
  - **PyDSTool.Toolbox.optimizers.line\_search.barzilai\_borwein\_search**
  - **PyDSTool.Toolbox.optimizers.line\_search.cubic\_interpolation**: *Line Search with the cubic interpolation method with the
    computation of the gradient of the function*
  - **PyDSTool.Toolbox.optimizers.line\_search.damped\_line\_search**: *A damped line search*
  - **PyDSTool.Toolbox.optimizers.line\_search.fibonacci\_section**: *Line Search with the Fibonacci section method*
  - **PyDSTool.Toolbox.optimizers.line\_search.fixed\_last\_step\_modifier**: *Line search decorator that overrides the default alpha\_step value
    with a factor times the last alpha\_step*
  - **PyDSTool.Toolbox.optimizers.line\_search.golden\_section**: *Line Search with the golden section method*
  - **PyDSTool.Toolbox.optimizers.line\_search.goldstein\_rule**
  - **PyDSTool.Toolbox.optimizers.line\_search.hyperbolic\_line\_search**: *An hyperbolic line search, in fact no searches at all*
  - **PyDSTool.Toolbox.optimizers.line\_search.quadratic\_interpolation**: *Line Search with the quadratic interpolation method with the
    computation of the gradient of the function*
  - **PyDSTool.Toolbox.optimizers.line\_search.scaled\_line\_search**
  - **PyDSTool.Toolbox.optimizers.line\_search.simple\_line\_search**: *A simple line search, in fact no searches at all*
  - **PyDSTool.Toolbox.optimizers.line\_search.strong\_wolfe\_powell\_rule**
  - **PyDSTool.Toolbox.optimizers.line\_search.wolfe\_powell\_rule**
- **PyDSTool.Toolbox.optimizers.optimizer**: *Module containing the core optimizers*
  - **PyDSTool.Toolbox.optimizers.optimizer.optimizer**: *The core optimizer from which every other optimizer is derived*
  - **PyDSTool.Toolbox.optimizers.optimizer.standard\_optimizer**: *A standard optimizer*
  - **PyDSTool.Toolbox.optimizers.optimizer.standard\_optimizer\_modifying**: *A standard optimizer with a special object that modifies the
    resulting set of parameters*
- **PyDSTool.Toolbox.optimizers.step**: *Module containing every step use to lower a cost function*
  - **PyDSTool.Toolbox.optimizers.step.conjugate\_gradient\_step**: *Computes the conjugate gradient steps for a specific function at a
    specific point*
  - **PyDSTool.Toolbox.optimizers.step.goldfeld\_step**: *Computes Goldfeld step for a specific function at a specific point*
  - **PyDSTool.Toolbox.optimizers.step.goldstein\_price\_step**: *Computes Goldstein-Price step for a specific function at a specific
    point*
  - **PyDSTool.Toolbox.optimizers.step.gradient\_step**: *Computes a gradient step for a specific function at a specific
    point*
  - **PyDSTool.Toolbox.optimizers.step.local\_brute\_force\_1dstep**
  - **PyDSTool.Toolbox.optimizers.step.marquardt\_step**: *Computes a Marquardt step for a specific function at a specific
    point*
  - **PyDSTool.Toolbox.optimizers.step.newton\_step**: *Computes a Newton step for a specific function at a specific point*
  - **PyDSTool.Toolbox.optimizers.step.partial\_step**: *Computes a partial step for a specific function at a specific
    point, acting like a decorator for other steps*
  - **PyDSTool.Toolbox.optimizers.step.quasi\_newton\_step**: *Computes a quasi-Newton step for a specific function at a specific
    point*
  - **PyDSTool.Toolbox.optimizers.step.restart\_conjugate\_gradient**: *Restarts a conjugate gradient search by deleting the step key in
    the state dictionary*
- **PyDSTool.Toolbox.optimizers.tests**
  - **PyDSTool.Toolbox.optimizers.tests.test\_powell**: *Class defining the Powell function*
  - **PyDSTool.Toolbox.optimizers.tests.test\_quadratic**: *Class defining a quadratic function*
  - **PyDSTool.Toolbox.optimizers.tests.test\_rosenbrock**: *Class defining the Rosenbrock function*

  


|  |  |
| --- | --- |
| Functions | [hide private] |

|  |  |
| --- | --- |
| mod\_path() | source code |

|  |  |
| --- | --- |
| test(level=-5, verbosity=1) | source code |

|  |  |
| --- | --- |
| testall(level=-5, verbosity=1) | source code |

| Home | Trees | Indices | Help | PyDSTool |
| --- | --- | --- | --- | --- |

|  |  |
| --- | --- |
| Generated by Epydoc 3.0.1 on Fri May 4 15:24:05 2012 | http://epydoc.sourceforge.net |
